# Two-Stage pH Control Strategy Based on the pH Preference of Acetoin Reductase Regulates Acetoin and 2,3-Butanediol Distribution in *Bacillus subtilis*

**DOI:** 10.1371/journal.pone.0091187

**Published:** 2014-03-07

**Authors:** Xian Zhang, Teng Bao, Zhiming Rao, Taowei Yang, Zhenghong Xu, Shangtian Yang, Huazhong Li

**Affiliations:** 1 The Key Laboratory of Industrial Biotechnology of Ministry of Education, School of Biotechnology, Jiangnan University, Wuxi, Jiangsu, P. R. China; 2 School of Medicine and Pharmaceuticals, Jiangnan University, Wuxi, Jiangsu, P. R. China; 3 Department of Chemical Engineering, Ohio State University, Columbus, Ohio, United States of America; Institut Pasteur Paris, France

## Abstract

Acetoin reductase/2,3-butanediol dehydrogenase (AR/BDH), which catalyzes the interconversion between acetoin and 2,3-butanediol, plays an important role in distribution of the products pools. This work characterized the *Bacillus subtilis* AR/BDH for the first time. The enzyme showed very different pH preferences of pH 6.5 for reduction and pH 8.5 for oxidation. Based on these above results, a two-stage pH control strategy was optimized for acetoin production, in which the pH was controlled at 6.5 for quickly converting glucose to acetoin and 2,3-butanediol, and then 8.0 for reversely transforming 2,3-butanediol to acetoin. By over-expression of AR/BDH in the wild-type *B. subtilis* JNA 3-10 and applying fed-batch fermentation based on the two-stage pH control strategy, acetoin yield of *B. subtilis* was improved to a new record of 73.6 g/l, with the productivity of 0.77 g/(l·h). The molar yield of acetoin was improved from 57.5% to 83.5% and the ratio of acetoin/2,3-butanediol was switched from 2.7∶1 to 18.0∶1.

## Introduction

Acetoin, naturally in fruits, corn, meet and some fermented food, is a famous spice that can be used to add flavor to food. It can also be used in cosmetics and chemical synthesis. Fermentation of acetoin by microorganisms is favorable since the process can use cheap substrate and has less environmental stresses. The vital physiological significance of acetoin to microorganisms is mainly in avoiding acidification, participating in the regulation of NADH/NAD^+^ ratio and storing carbon [1]. With the increasing demand of food flavor, nature acetoin produced by microbial fermentation is popular.

**Figure 1:**
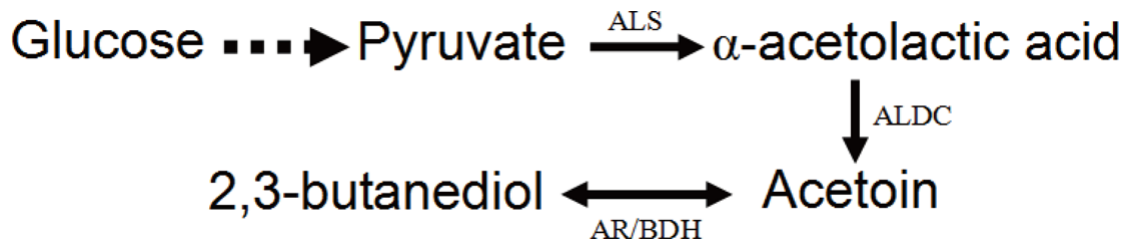
Acetoin metabolic pathway. ALS: α-acetolactic acid synthase; ALDC: α-acetolactic acid decarboxylase; AR/BDH: acetoin reductase/2,3-butanediol dehydrogenase.

Many species such as Saccharomyces cerevisiae [2], Leuconostoc mesenteroides [3], Enterobacter aerogenes [4], Bacillus subtilis [5], Serratia marcescens [6], Lactococcus lactis [7], Klebsiella oxytoca [8] and Paenibacillus polymyxa [9] can be used to produce acetoin. However, in many cases, acetoin is only a byproduct of 2,3-butanediol during the fermentation, which is also an very important chemical [10]. Among the strains mentioned above, Bacillus species, on the Food and Drug Administration's GRAS (generally regarded as safe) list, have been developed and engineered as industrial producers of nucleotides, the vitamin riboflavin, the flavor agent ribose, and the supplement poly-gamma-glutamic acid [11]. With the characterization of B. subtilis genome, the species is poised to become a preferred host for the production of many new and improved products [12]. Our lab isolated a B. subtilis strain, which produced 42.2 g/l acetoin and 15.8 g/l 2,3-butanediol in about 132 h. The strain could reversely transform 2,3-butanediol to acetoin in the decline phase of fermentation by the enzyme acetoin reductase/2,3-butanediol dehydrogenase (AR/BDH EC 1.1.1.4) [5] (Figure 1).

AR/BDH, also named diacetyl reductase, catalyses both the reversible oxidation of 2,3-butanediol to acetoin and the practically irreversible reduction of diacetyl to acetoin [13], [14]. The enzyme plays an important role in distribution of acetoin and 2,3-butanediol proportions as well as NADH/NAD^+^ pools. It has been purified and characterized from several microorganisms. AR/BDH has very strict cofactor specificity and can only utilize NADH for reduction and NAD^+^ for oxidation. A very special property of AR/BDH has been reported that it has different optimum pH-values for oxidation and reduction, respectively. Table 1 shows the specific optimum pH values of AR/BDH from *Bacillus polymyxa*, *Serratia marcescens*, *Gluconobacter oxydans* [15], *Lactococcus lactis* [16], *Saccharomyces cerevisiae* [17]-[19], *Pyrococcus furiosus* [20] and *Rhodococcus erythropolis* WZ010 [21]. The results indicate that the enzyme AR/BDH preferentially catalyzes the reduction/oxidation reaction in the acidic/alkaline condition.

**Table 1:** The optimum pH values of AR/BDH from different microorganisms.

Strains	Optimum pH for Reduction	Optimum pH for oxidation
*Bacillus polymyxa* [15]	7.0	9.0
*Serratia marcescens* [15]	4.5	9.0
*Gluconobacter oxydans* [15]	7.0	9.0
*Lactococcus lactis* [16]	5.8	10.0
*Saccharomyces cerevisiae* [17]	6.7	7.2
*Saccharomyces cerevisiae* [18], [19]	7.0	8.0
*Pyrococcus furiosus* [20]	6.1	8.8
*Rhodococcus erythropolis WZ010* [21]	7.0	9.5

Recently, for improving acetoin production, efforts have been focused on screening new bacterial strains [8], [22]-[24] and optimizing the fermentation mediums [25], [26]. New findings have also proved that the fermentation duration can be shortened effectively and acetoin production can be improved by changing the agitation speed to control the dissolved oxygen levels [23], [27]. However, there was still considerable amount of byproduct 2,3-butanediol through these above efforts. The similar problem existed in 2,3-butanediol producers that acetoin was a major byproduct in the fermentation. These byproducts produced by the catalysis of AR/BDH caused the loss of substrate and energy. Science AR/BDH plays such a critical role in distribution of acetoin and 2,3-butanediol proportions, the reversal reaction by this enzyme restrains the improvement of acetoin production. Therefore, besides screening of new bacterial strains, optimizing the culture mediums and controlling the dissolved oxygen levels, the conditions for AR/BDH reaction during fermentation should have the priority to be studied. The *B. subtilis* AR/BDH, encoded by the *bdhA* gene [28], had been over-expressed in *Escherichia coli* BL21 [29]. But the enzyme has never been purified and characterized, which limits the optimum use of *B. subtilis* for acetoin or 2,3-butanediol production.

In this work, the AR/BDH from *B. subtilis* JNA 3–10 was cloned and overexpressed, and its properties were studied for the first time. Based on pH preferences of AR/BDH, the two-stage pH control strategy was proposed to redistribute acetoin and 2,3-butanediol proportions. With the optimum pH control strategy for acetoin production, the recombinant *B. subtilis* overexpressing AR/BDH was used to yield abundant amount of acetoin and decrease the yield of byproduct 2,3-butanediol.

## Materials and Methods

### Strains, plasmids and primers

The strains, plasmids and primers used in this study were listed in Table 2.

**Table 2:** Bacterial strains, plasmids and primers used.

Strains/plasmids/primers		Characteristics	Sources
Strains			
*E. coli* JM109	*recA1, endA1, gyrA96, thi-1, hsd R17(r_k_^−^ m_k_^+^)supE44*		Invitrogen
*B. subtilis* JNA 3–10	Wild type strain		Laboratory stock
BSA	*B. subtilis* JNA 3–10 containing pMA5-*bdhA* (Km^R^)		This study
Plasmids			
pMA5	*Hpa*II, colE1, *repB*, replicates in *E. coli* (Amp^R^) or *B. subtilis* (Km^R^)		Laboratory stock
pMA5-*bdhA*	pMA5 containing *bdhA*-His		This study
Primers 5′-3′			
PF	ACCGGGATCCATGAAGGCAGCAAGATGG (*Bam*H I)		
PR	ACCGACGCGTTTAGTGGTGGTGGTGGTGGTGGTTAGGTCTAACAAGG (*Mlu* I)		

Amp^R^ ampicillin-resistant, Km^R^ kanamycin-resistant, the restriction enzyme sites were bold typed, and the His-Tag coding region was underlined.

### Preparation of crude enzyme and AR/BDH purification

The recombinant *B. subtilis* was cultured in Luria-Bertain (LB) medium at 37°C on a rotary shaker at 160 rpm. After cultured for 24 h, cells were collected by centrifugation at 8000 rpm for 30 min at 4°C. The cell pellets were suspended and washed with 0.1 M potassium phosphate buffer (pH 7.0) for there times. For preparation of crude AR/BDH, the cells were resuspended in 0.1 M potassium phosphate buffer containing 0.1 mM β-Mercaptoethanol and 2 µg/ml PMSF, pH 6.5. Crude enzyme was prepared by sonication after treated with 1 mg/ml lysozyme for 60 min at 4°C. The homogenate was centrifuged at 15,000 rpm for 60 min at 4°C. The activity of AR/BDH was detected as described previously [28].

The recombinant AR/BDH was expressed as a His_6_-tagged protein in *B. subtilis* JNA 3–10. The recombinant protein was purified by affinity chromatography on a Ni-NTA agarose prepacked column HisTrap HP (GE Healthcare, Uppsala, Sweden). The pooled fractions were then loaded on a Superdex™ 200 (10/300 GL) equilibrated with the buffer (20 mM Tris-HCl and 150 mM NaCl, pH 8.0) using an ÄKTA Protein Purifier system (Pharmacia, Uppsala, Sweden). The enzyme was assayed by sodium dodecyl sulfate-polyacrylamide gel electrophoresis (SDS-PAGE) according to Laemmli method [30]. The molecular weight of protein was determined by comparing the relative mobility of perfect protein Marker 14.4–116 kDa (Thermo, USA). The protein concentration was determined by Bradford method [31] using BSA as the standard protein.

### Effect of pH and temperature on enzyme activity and stability

The following buffer systems were used to investigate the pH dependence of AR/BDH: 50 mM sodium acetate buffer (pH 4.5∼6.0), 50 mM phosphate buffered saline buffer (PBS buffer, pH 6.0∼8.0) and 50 mM glycine-NaOH buffer (pH 8.0∼10.5). The spontaneous oxidation of NADH in buffers of low pH was corrected when detecting the effect of pH on enzyme activity. The activity of AR/BDH was assayed at different temperatures from 25°C to 60°C with a gradient of 5°C. The enzyme stability was investigated using the standard AR/BDH assay after it had been incubated for 2 h under different conditions.

### Effect of chemical inhibitors and stimulators on enzyme activity

The influence of a variety of mental ions and EDTA on enzyme activity was determined. The reaction mixture containing 100 ng enzyme in 50 mM PBS buffer (pH 6.5 for reduction activity and pH 8.5 for oxidation activity) were incubated in the appropriate chemical for 20 min at 25°C. After preincubation, NADH/NAD^+^ (final concentration 0.2 mM/1.2 mM) was added and AR/BDH activity was measured with 10 mM acetoin/2,3-butanediol as substrate. The following chemicals were assayed at a final concentration of both 1 mM and 3 mM: NaCl, KCl, MgSO_4_, MnSO_4_, ZnCl_2_, CaCl_2_, CuSO_4_ and EDTA.

### Substrate specificity and kinetics properties

The oxidation/reduction of NADH/NAD^+^ and NADPH/NADP^+^ with the consumption of acetoin/2,3-butanediol were measured under standard assay conditions described for AR/BDH, to test if these pyridine nucleotides could act as alternative electron donors/accepters. The *K*_m_ and *V*_max_ values were determined from Lineweaver-Burk plots of the data.

### Culture conditions

*B. subtilis* and *Escherichia coli* were cultured in Luria-Bertani (LB) medium at 37°C on a rotary shaker at 160 rpm. When necessary, the LB medium was supplemented with 100 µg/ml ampicillin or 50 µg/ml kanamycin. For acetoin fermentation, the cells were inoculated into 10 ml LB medium, cultivated for 7–9 h, and then 1 ml of the culture was transferred into 50 ml seed medium (LB with 40 g/L glucose) for preparing the seed culture. After 10 h, 2.5 ml of the seed culture (OD_600_ = 5.0–6.0) was inoculated into 50 ml of fermentation medium (glucose 100 g/l, beef extract 5 g/l, corn steep liquor 20 g/l, and urea 2 g/l). When fermentation carried out in a 5-L fermentor (Biotech Co., Shanghai, China), the agitation speed was set at 300 rpm.

### Analytical methods

Residual glucose was detected using a SBA-40C biological sensing analyzer (SBA, China). Acetoin and 2,3-butanediol were detected by gas chromatography (Jie Dao GC1600, China, FID detector, N_2_ flow rate of 50 ml/min, detector temperature of 250°C, and a column temperature of 160°C). Biomass was measured spectrophotometrically at 600 nm (UNICO UV-2000, USA). All assays were performed in duplicate or triplicate.

## Results and Discussion

### Expression and purification of AR/BDH

**Figure 2:**
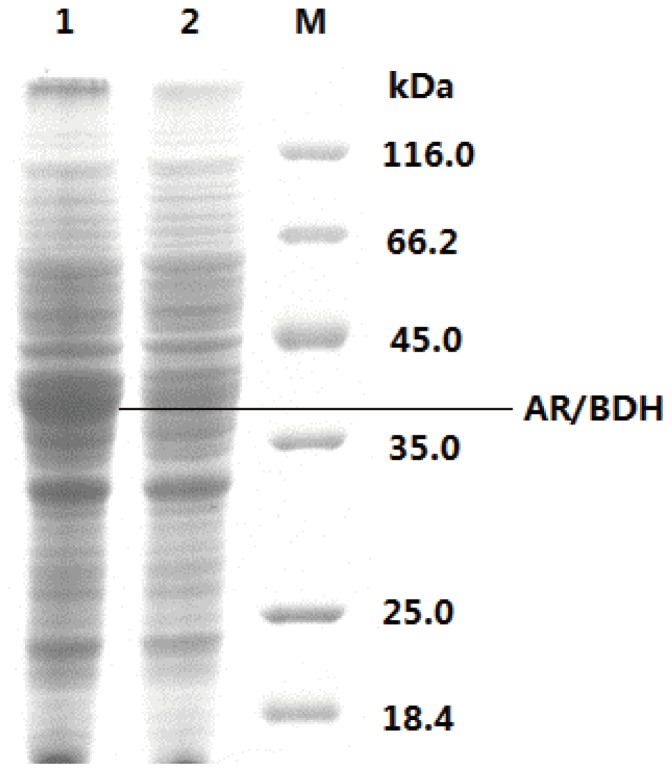
SDS-PAGE analysis of AR/BDH expression. Lanes 1: BSA; 2: *B. subtilis* JNA 3–10; M: molecular mass standards.

**Figure 3:**
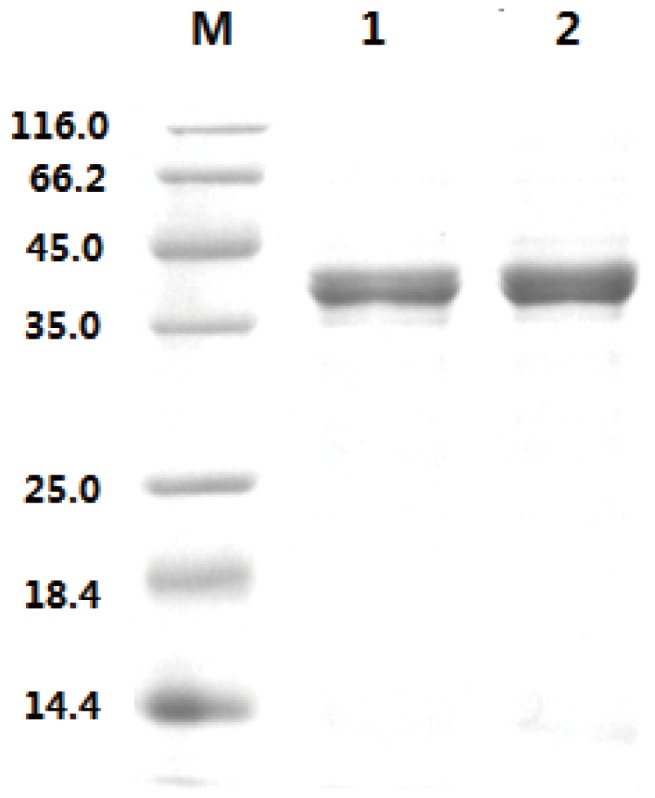
SDS-PAGE analysis of AR/BDH purification. Lanes 1, 2: AR/BDH; M: molecular mass standards.

Plasmid pMA5-*bdhA* was constructed and transformed into *B. subtilis* JNA 3–10, resulting in BSA strain. After cultured for 24 h, the cells of BSA strain were collected for preparation of crude AR/BDH. The purification of the *B. subtilis* AR/BDH relies on affinity binding of enzyme with His-tag by Ni^2+^. The apparent subunit molecular mass of the AR/BDH was about 38-39 kDa as determined by SDS-PAGE (Figure 2). The protein was purified about 18-fold and has a specific activity of 140.9 mU/mg (Figure 3).

### BSATemperature and pH dependence of *B. subtilis* AR/BDH

**Figure 4:**
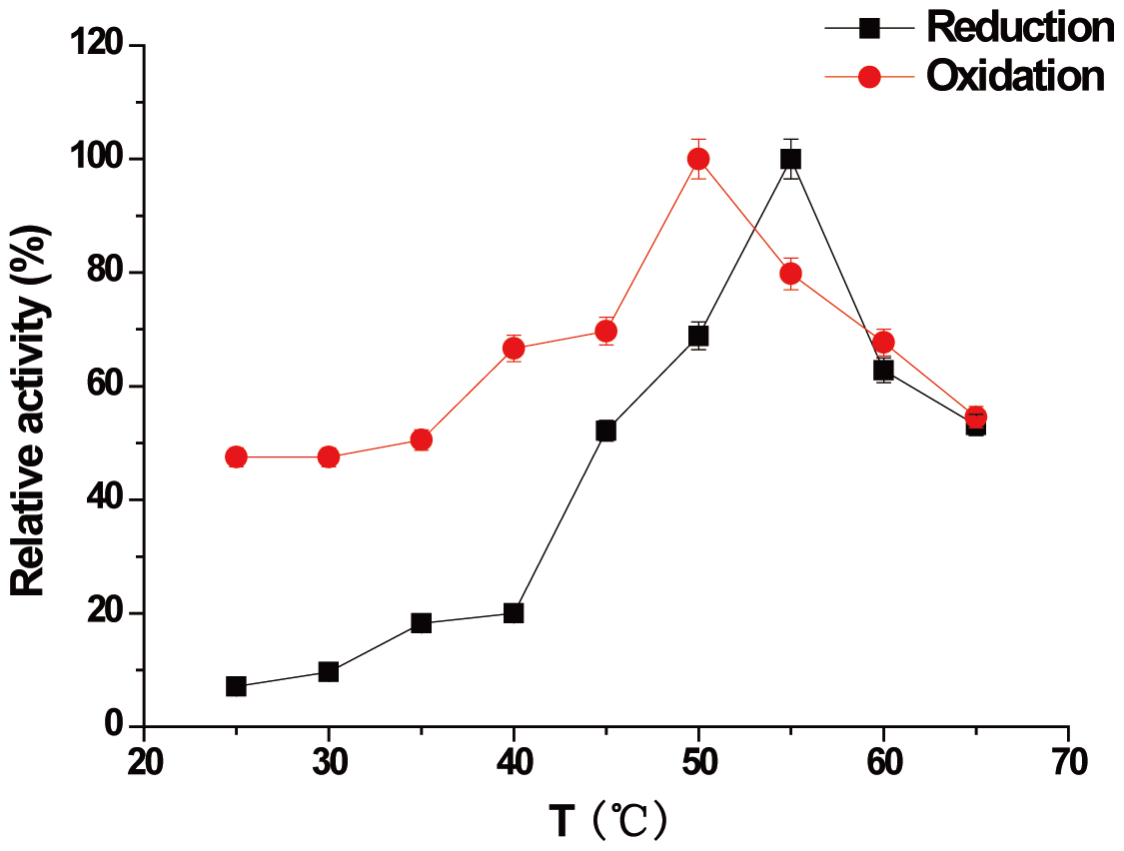
Effect of temperature on AR/BDH activity. Reduction (AR); Oxidation (BDH).

**Figure 5:**
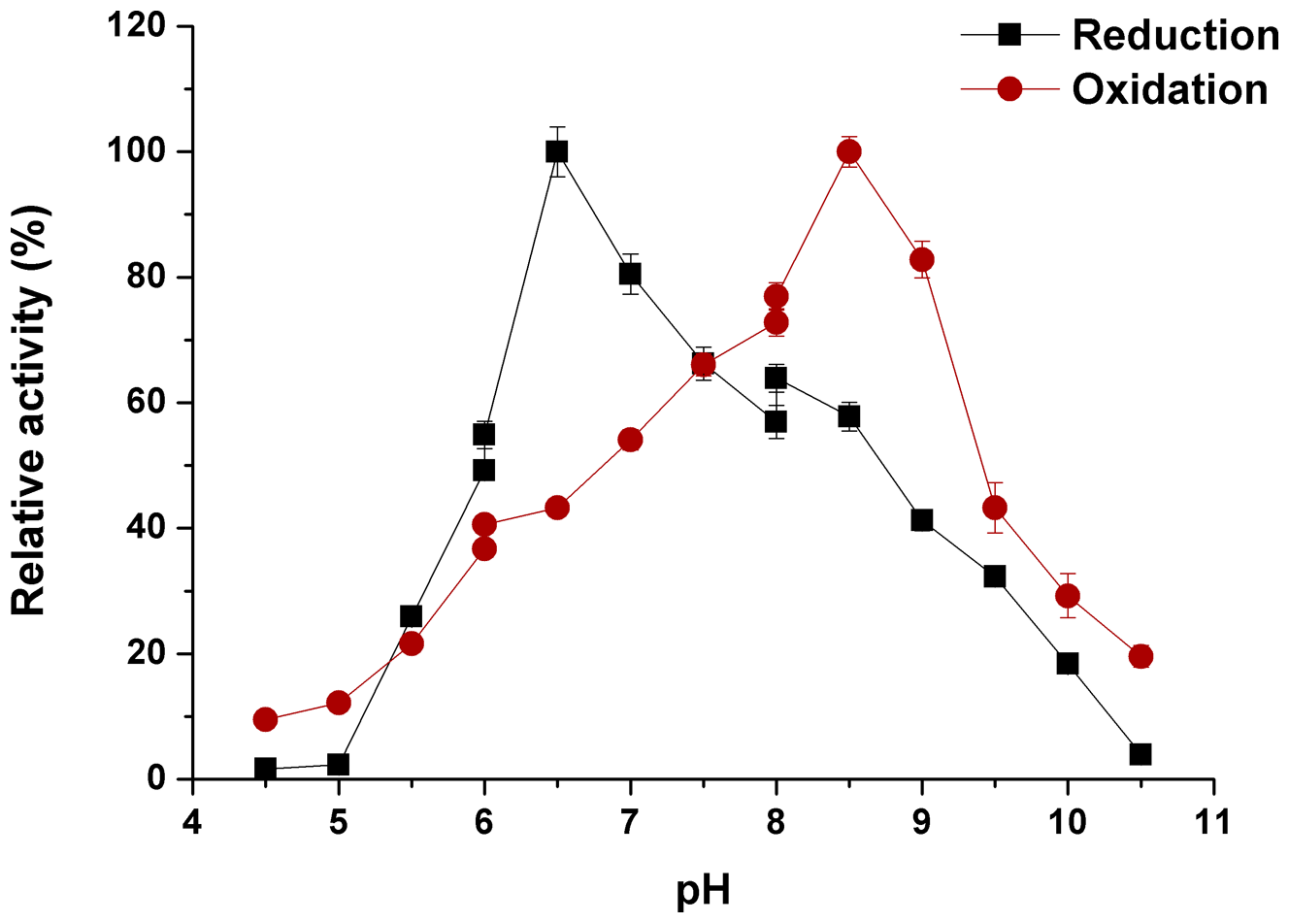
Effect of pH on AR/BDH activity. Reduction (AR); Oxidation (BDH).

The optimum temperature for AR/BDH activity was about 55°C/50°C (Figure 4). However, the enzyme showed very different optimum pH dependences on the reduction and oxidation reactions, which were pH 6.5 and pH 8.5 respectively (Figure 5). The enzyme was very unstable when stored above 20°C. After incubated at 0°C for 2 hours, the enzyme maintained only 80% activity.

### strain Chemical inhibitors and stimulators

Of all the chemicals listed in Table 3, we investigated that 3 mM Mn^2+^ greatly stimulated the activity of BDH, while Zn^2+^ and Cn^2+^ had inhibitive effects on both AR and BDH activities. Ca^2+^ showed a weak activation effect on BDH activity but no effect on AR activity. The other metals and EDTA did not enhance or inhibit AR and BDH activities obviously (within the range of ± 15%).

**Table 3:** Effect of mental ions and EDTA on enzyme activities.

Chemicals	AR relative activity (%)		BDH relative activity (%)	
	1 mM	3 mM	1 mM	3 mM
Na^+^	99.4±2.3	95.1±1.2	110.3±3.2	113.3±5.1
K^+^	98.2±1.5	100.4±0.9	105.3±1.3	88.6±1.8
Mg^2+^	93.6±3.1	96.4±2.4	124.8±2.1	133.9±2.9
Mn^2+^	111.8±2.8	112.6±3.1	144.5±3.1	252.3±5.8
Zn^2+^	10.8±2.1	7.7±0.4	15.5±0.8	0
Ca^2+^	106.1±1.9	102.3±1.5	136.6±2.5	114.3 ±1.1
Cu^2+^	31.5±0.8	23.1±0.5	21.3±0.8	0
EDTA	100.7±1.8	101.9±2.1	102.5±1.6	103.3±1.3

### Substrate specificity and kinetics properties

Under standard assay condition described for AR/BDH, the NADH/NAD^+^ was directly oxidized/reduced while NADPH/NADP^+^ was not detectably oxidized/reduced by the enzyme. The *K*_m_ and *V*_max_ values for NADH/NAD^+^ and acetoin/2,3-butanediol were given in Table 4. The catalytic efficiency constant, *k*_cat_/*K*_m_, were greater for the reduction of acetoin than for the oxidation of 2,3-butanediol, indicating the enzyme could preferentially function as a reductase rather than as a dehydrogenase.

**Table 4:** Kinetic constants of *B. subtilis* AR/BDH.

Substrate	*K*_m_ (mmol/l)	*V*_max_ (μmol/l·min)	*k*_cat_ (1/min)	*k*_cat_/*K*_m_ (l/min·mmol)
Acetoin	0.16±0.005	5.54±0.3	0.34±0.012	2.13±0.2
2,3-butanediol	0.26±0.009	2.34±0.1	0.14±0.005	5.40±0.5
NADH	0.04±0.001	14.2±0.7	1.41±0.021	3.53±0.2
NAD^+^	0.08±0.002	14.0±0.7	1.39±0.016	1.74±0.1

### Effect of initial pH on acetoin fermentation

**Figure 6:**
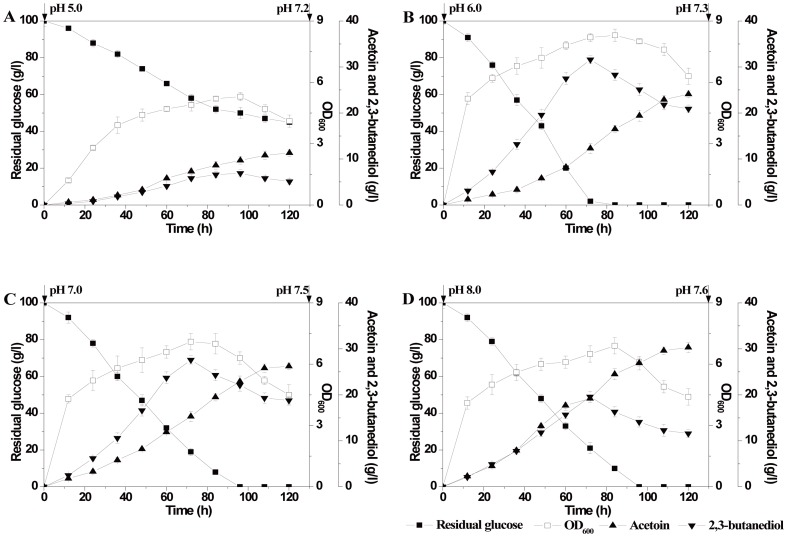
Effect of initial fermentation pH on *B. subtilis* JNA 3-10 for acetoin production.

In previous shake flask fermentation, the fermentation medium was not adjusted with nature pH (after autoclaved sterilization, the medium pH was about 6.2∼6.4). *B. subtilis* JNA 3–10 could completely consume 100 g/l glucose in about 72 h accompanied by rapid accumulation of 2,3-butanediol, then part of 2,3-butanediol was reversely transformed into acetoin. At about 120 h, 25.2 g/l acetoin and 19.8 g/l 2,3-butanediol could be obtained. Since the optimum pH of AR and BDH have very different preferences, this work studied the effect of the initial pH (5.0, 6.0, 7.0 and 8.0) of the medium on *B. subtilis* JNA 3–10 fermentation in shake flask (Figure 6). The results indicated that fermentation pH played a vital role in acetoin/2,3-butanediol proportion.

#### Cell growth and glucose consumption rate

The results showed that glucose consumption rate was coupled with cell growth. When the initial fermentation pH was 5.0, cell growth was inhibited greatly and was accompanied by very slowly glucose consumption rate (Figure 6 A). The highest glucose consumption rate was observed when initial fermentation pH was 6.0 (Figure 6 B), about 100 g/l glucose was consumed within 72 h, suggesting faintly acid environment was favorable for *B. subtilis* growth. However, when the initial fermentation pH was equal or higher than 9.0, the cells can not growth (data was not shown).

#### Acetoin and 2,3-butanediol production

When the initial fermentation pH was 6.0, with the highest glucose consumption rate, the acetoin production was the lowest compared whit that of pH 7.0 (Figure 6 C) and pH 8.0 (Figure 6 D). When the initial fermentation pH was raised artificially, the efficiency of 2,3-butanediol production was decreased, because the tendency of the reversible reaction between acetoin and 2,3-butanediol was moving in the direction of BDH.

At the end of fermentation, the culture pH reached to about the same level, suggesting *B. subtilis* strain can adjust its own acid-base equilibrium by fermentation. Therefore, in decline phase of fermentation, there were little differences in the transformation rates from 2,3-butanediol to acetoin. We also observed that the reversal of AR/BDH reaction only occurred when fermentation just entered the late stationary phase or the decline phase. The results strongly recommended that controlling of the pH that favorable for BDH activity while fermentation entered the late stationary phase could further increase acetoin production.

### Two-stage pH control strategies in 5-L fermentor

**Figure 7:**
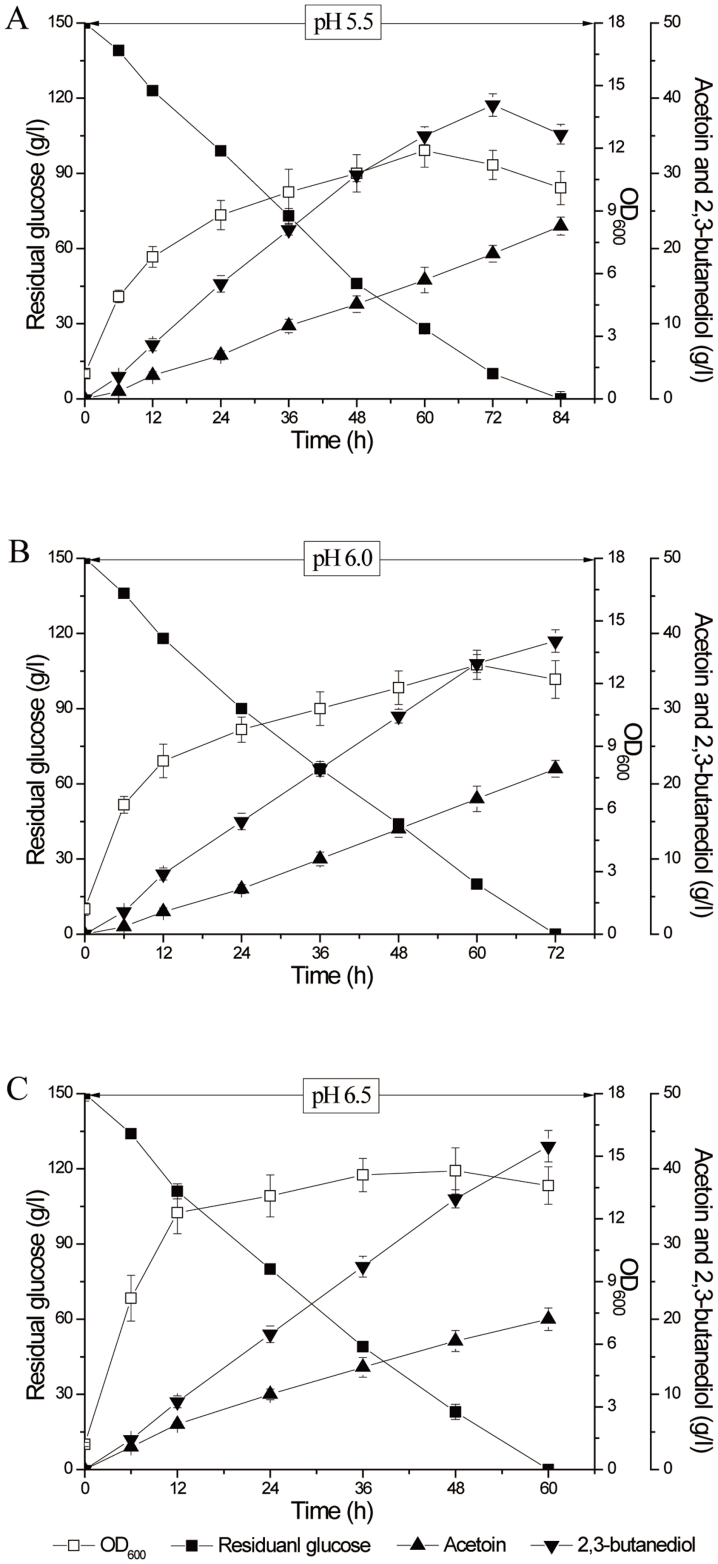
Timer course of acetoin fermentation by *B. subtilis* JNA 3-10 in 5-L fermentor. The fermentation was under different pH and was terminated on the depletion of glucose. A (pH 5.5); B (pH 6.0); C (pH 6.5).

**Figure 8:**
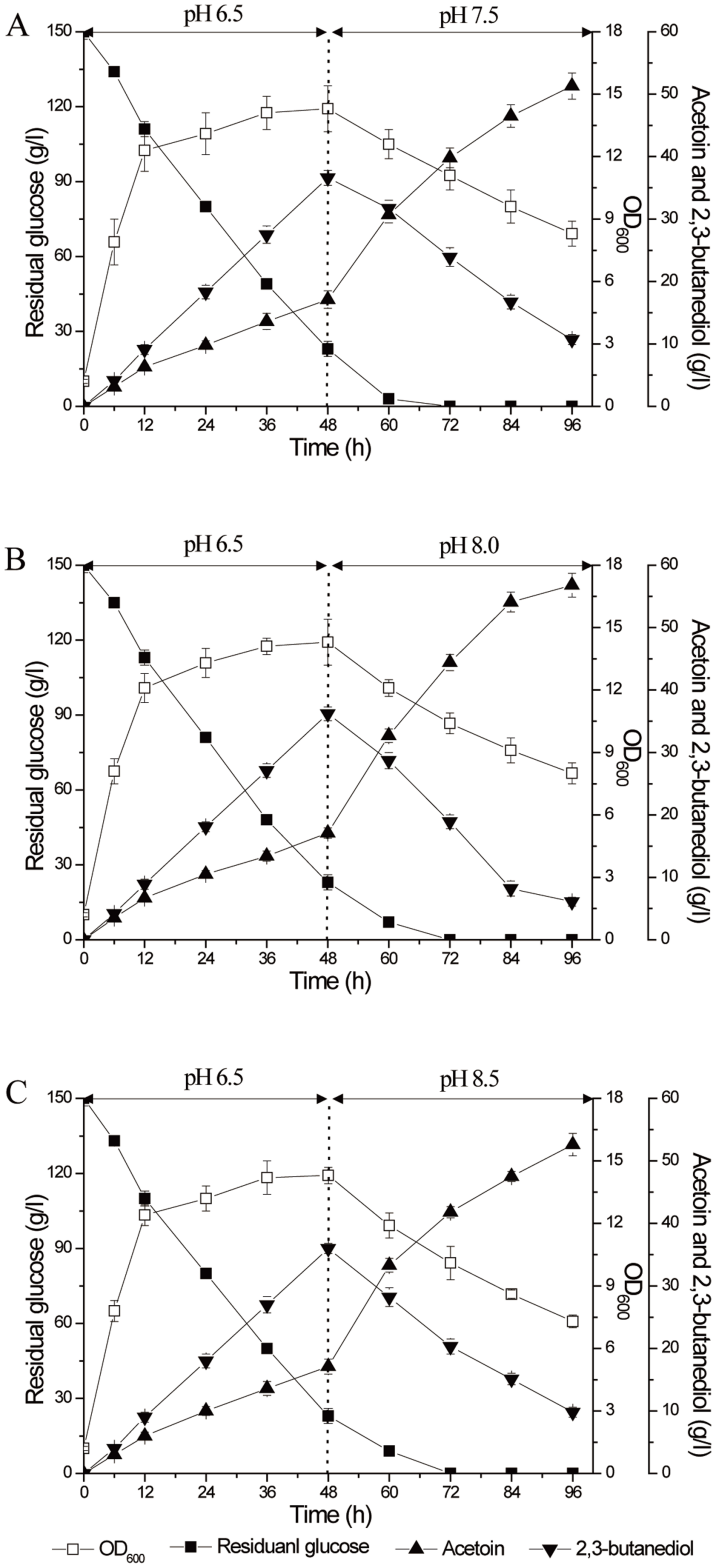
Timer course of acetoin fermentation by *B. subtilis* JNA 3-10 in 5-L fermentor using two-stage pH control strategies. A (pH 6.5 for the first 48 h and pH 7.5 for the last 48 h); B (pH 6.5 for the first 48 h and pH 8.0 for the last 48 h); C (pH 6.5 for the first 48 h and pH 8.5 for the last 48 h).

The two-stage pH control strategy was proposed based on comprehensive consideration of the cell growth, glucose consumption rate and AR/BDH activity. In a 5-L fermentor, the fermentation pH was first controlled around 6.0 (5.5, 6.0 and 6.5), which was supposed to quickly consume glucose (Figure 7). The results demonstrated that 150 g/l glucose was totally consumed in 60 h at pH 6.5, and the biomass was the highest in this condition. Once the cells fermented into the late stationary phase, 2,3-butanediol was reversely transformed into acetoin. Then we proposed to speed the reverse transformation process at about 48 h by switching the fermentation pH from 6.5 to around 8.0 (7.5, 8.0 and 8.5) (Figure 8). The results showed that pH 8.0 was favorable for quickly transforming 2,3-butanediol to acetoin in the fermentation. Thought the optimum pH for BDH oxidation was 8.5, the biomass decreased greatly and the enzyme inactivated quickly at that pH. Thus, the optimum strategy for the two-stage pH control fermentation was proposed at pH 6.5 in the first 48 h and pH 8.0 at the last 48 h (Figure 8 B). Using this strategy, 150 g/l glucose was converted into 56.8 g/l acetoin and 6.1 g/l 2,3-butanediol. Acetoin productivity was improved to 0.59 g/(l·h), about 84% higher than described previously (produced 42.2 g/l acetoin in 132 h, with acetoin molar yield of 57.5% and productivity of 0.32 g/(l·h)) [5].

### B Highly improved acetoin production by over-expression of the AR/BDH

**Figure 9:**
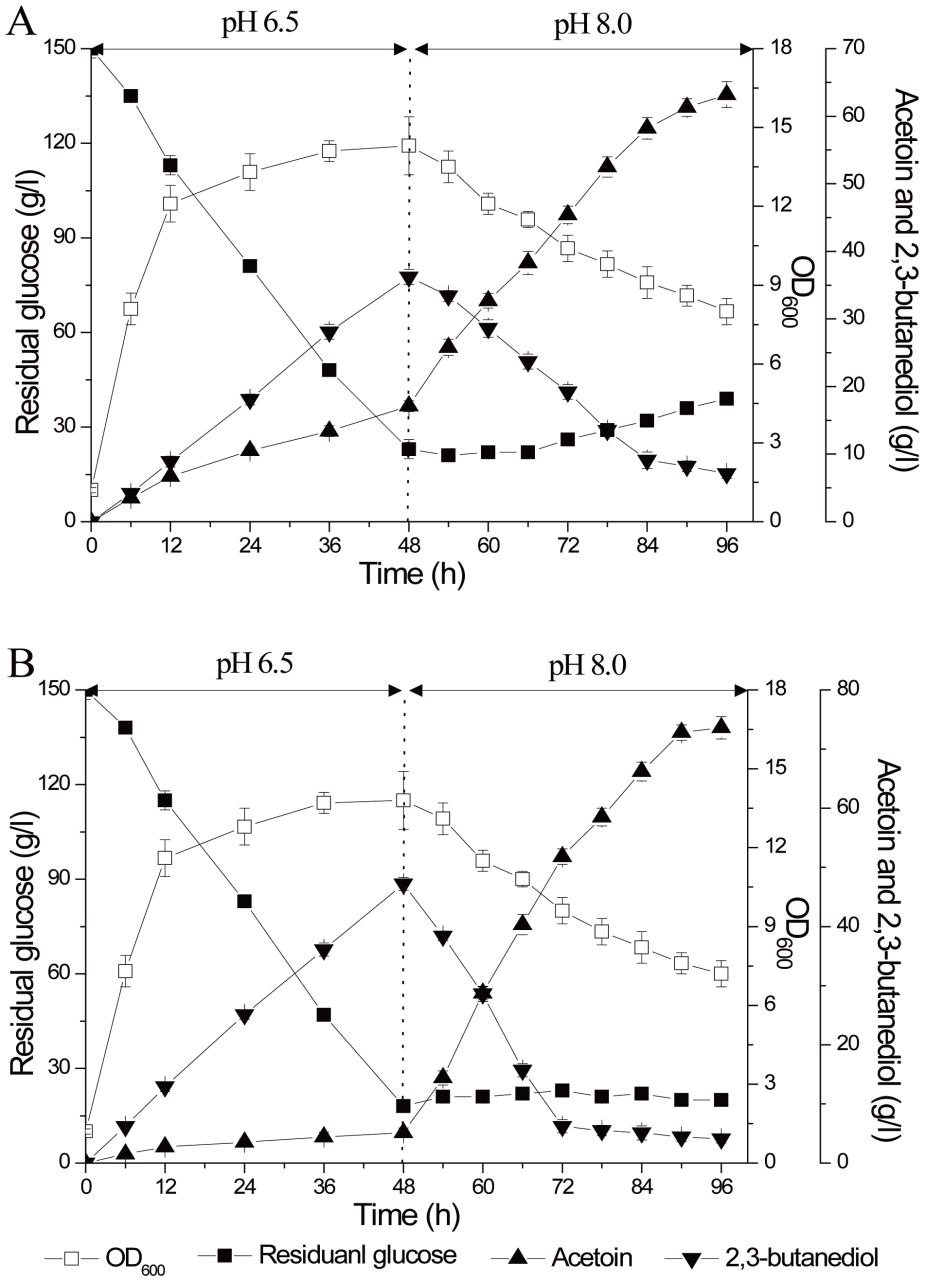
Fed-batch fermentation of strain JNA 3-10 and BSA in 5-L fermentor based on the optimum two-stage pH control strategy. A (strain JNA 3–10); B(strain BSA).

To investigate the significant role of AR/BDH in redistribution of the metabolic flux between acetoin and 2,3-butanediol. The recombinant strain BSA was then fermented based on the optimum two-stage pH control strategy. In the two-stage pH control fermentation described above, glucose was totally consumed in about 60 h. Thus, fed-batch fermentation was proposed during the second stage of fermentation, in which the pH was controlled at 8.0 (Figure 9).

By over-expression of AR/BDH, the transformation rate between acetoin and 2,3-butanediol was obviously increased (Figure 9 B) compared to JNA 3-10 (Figure 9 A). In the first stage of fermentation, acetoin was immediately transformed to 2,3-butanediol after it was synthesized from α-acetolactic acid. As soon as the fermentation pH was switched to 8.0, 2,3-butanediol was reversely transformed to acetoin. Almost all of the byproduct 2,3-butanediol was transformed to acetoin by the recombinant strain (4.1 g/l left) compare to JNA 3-10 (7.1 g/l left). While the yield of 2,3-butanediol was decreased to a low level, it can not be reversely transformed to acetoin, indicating other restrictive factors such as NADH/NAD^+^ [32] or dissolved oxygen levels [23], [27] may restrain the reversible reaction. In the second stage of fermentation, acetoin was quickly accumulated not only because of the reversible enzymatic reaction but also the conversion from glucose. The over-expression of AR/BDH enhanced the consumption rate of glucose, and JNA 3–10 and BSA consumed about 163 g/l and 180 g/l glucose, respectively, which corresponding to the total acetoin and 2,3-butanediol production of 70.3 g/l and 77.7 g/l. By applying over-expression of AR/BDH and fed-batch fermentation based on the two-stage pH control strategy, acetoin production was improved to 73.6 g/l. By consumption of about 180 g/l glucose, acetoin molar yield was 83.5%, with a productivity of 0.77 g/(l·h). To our knowledge, it is the highest record of acetoin production in the GRAS *B. subtilis*.

## Conclusions

*B. subtilis* AR/BDH was characterized in this work for the first time, and the recombinant enzyme was found to have optimum pH values of 6.5 for reduction and 8.5 for oxidation. A two-stage pH control strategy was proposed and optimized for acetoin production based on the pH preference of AR/BDH, in which the fermentation pH was controlled at 6.5 for the first 48 h and 8.0 for the last 48 h, respectively. By over-expression of AR/BDH and fed-batch fermentation based on the pH control strategy, acetoin yield was further improved to 73.6 g/l, which is the highest record of GRAS *B. subtilis*.
